# Protein synthesis inhibitors stimulate MondoA transcriptional activity by driving an accumulation of glucose 6-phosphate

**DOI:** 10.1186/s40170-020-00233-6

**Published:** 2020-12-04

**Authors:** Blake R. Wilde, Mohan R. Kaadige, Katrin P. Guillen, Andrew Butterfield, Bryan E. Welm, Donald E. Ayer

**Affiliations:** 1 Department of Oncological Sciences, Huntsman Cancer Institute, University of Utah, Salt Lake City, UT 84112 USA; 2 Present Address: Department of Biological Chemistry, David Geffen School of Medicine, University of California, Los Angeles, Los Angeles, CA 90095 USA; 3 Present Address: Translational Genomics Research Institute, Phoenix, AZ 85004 USA; 4 Department of Surgery, Huntsman Cancer Institute, University of Utah, Salt Lake City, UT 84112 USA

## Abstract

**Background:**

Protein synthesis is regulated by the availability of amino acids, the engagement of growth factor signaling pathways, and adenosine triphosphate (ATP) levels sufficient to support translation. Crosstalk between these inputs is extensive, yet other regulatory mechanisms remain to be characterized. For example, the translation initiation inhibitor rocaglamide A (RocA) induces thioredoxin-interacting protein (TXNIP). TXNIP is a negative regulator of glucose uptake; thus, its induction by RocA links translation to the availability of glucose. MondoA is the principal regulator of glucose-induced transcription, and its activity is triggered by the glycolytic intermediate, glucose 6-phosphate (G6P). MondoA responds to G6P generated by cytoplasmic glucose and mitochondrial ATP (mtATP), suggesting a critical role in the cellular response to these energy sources. TXNIP expression is entirely dependent on MondoA; therefore, we investigated how protein synthesis inhibitors impact its transcriptional activity.

**Methods:**

We investigated how translation regulates MondoA activity using cell line models and loss-of-function approaches. We examined how protein synthesis inhibitors effect gene expression and metabolism using RNA-sequencing and metabolomics, respectively. The biological impact of RocA was evaluated using cell lines and patient-derived xenograft organoid (PDxO) models.

**Results:**

We discovered that multiple protein synthesis inhibitors, including RocA, increase TXNIP expression in a manner that depends on MondoA, a functional electron transport chain and mtATP synthesis. Furthermore, RocA and cycloheximide increase mtATP and G6P levels, respectively, and TXNIP induction depends on interactions between the voltage-dependent anion channel (VDAC) and hexokinase (HK), which generates G6P. RocA treatment impacts the regulation of ~ 1200 genes, and ~ 250 of those genes are MondoA-dependent. RocA treatment is cytotoxic to triple negative breast cancer (TNBC) cell lines and shows preferential cytotoxicity against estrogen receptor negative (ER−) PDxO breast cancer models. Finally, RocA-driven cytotoxicity is partially dependent on MondoA or TXNIP.

**Conclusions:**

Our data suggest that protein synthesis inhibitors rewire metabolism, resulting in an increase in mtATP and G6P, the latter driving MondoA-dependent transcriptional activity. Further, MondoA is a critical component of the cellular transcriptional response to RocA. Our functional assays suggest that RocA or similar translation inhibitors may show efficacy against ER− breast tumors and that the levels of MondoA and TXNIP should be considered when exploring these potential treatment options.

**Supplementary Information:**

The online version contains supplementary material available at 10.1186/s40170-020-00233-6.

## Background

A unifying characteristic of oncogenes is their ability to drive anabolic metabolism to support the biosynthesis of macromolecules. Oncogenes also impose significant metabolic stress on cells [1]. For example, as a result of increased protein synthesis, cancer cells experience depletion of local nutrients [2], which can lead to accumulation of reactive oxygen species (ROS) and other metabolic challenges that if unchecked result in cell death [3]. These findings suggest that cells must integrate information about translation rate with the pathways that control nutrient availability. Recently, protein synthesis inhibitors have received attention as potential anticancer therapeutics [4–7], with translation initiation inhibitors among the most promising candidates. The full mechanistic and biological consequences of targeting translation initiation have not been described.

The translation inhibitor RocA induces expression of TXNIP in a number of cell types [6]; however, the underlying mechanisms were not explored. TXNIP has pleiotropic function [8, 9], including acting as a very potent negative regulator of glucose uptake [10]. Therefore, TXNIP may bridge translation initiation or elongation rate to the availability of glucose. TXNIP expression is strongly, if not entirely dependent on the MondoA transcription factor and glucose [10, 11]. Mechanistically, glucose 6-phosphate (G6P) drives translocation of MondoA from the outer mitochondrial membrane (OMM) to the nucleus where it binds the promoters of its target genes and recruits cofactors that initiate transcription [10, 12, 13]. MondoA binds a double E-box carbohydrate response element (ChoRE) in the TXNIP promoter to drive its expression in response to elevated glucose levels [11, 14, 15].

In addition to an absolute functional requirement on glucose [10, 11], we recently showed that MondoA transcriptional activity is also highly dependent on mtATP [13]. Our data suggest that MondoA functions as a coincidence detector, only being active when above threshold levels of glucose and mtATP are available to generate enough G6P to drive MondoA activity [13]. Collectively, our data suggest that MondoA is a sensor of high cellular energy charge exemplified by its two most prevalent nutrient sources and is critical for the adaptive transcriptional response to a hyper-nutrient state.

Here, we investigate whether MondoA is required for protein synthesis inhibitors to increase TXNIP expression. We provide evidence that protein synthesis inhibitors cause metabolic rewiring, resulting in increased levels of mtATP and G6P that drive MondoA transcriptional activity. Further, the cytotoxic effect of RocA depends on both MondoA and TXNIP, suggesting that they may be critically required for the utility of protein synthesis inhibitors in clinical settings.

## Methods

### Cell culture

All cell lines were maintained at 37 °C in 5% CO_2_. Dulbeccos Minimal Essential Media (DMEM) with penicillin/streptomycin and 10% fetal bovine serum (FBS) (Gibco) was used for murine embryonic fibroblasts (MEFs), HeLa, MDA-MB-231, L6, C2C12, and 293T (all from ATCC) and MDA-MB-157 cells (a gift from Andrea Bild, University of Utah). TSC2^−/−^ and TSC2^−/−^:hTSC2 MEFs were a gift of Brendan Manning, Harvard University. MondoA^−/−^ MEFs were created from day 15 embryos as described previously [16].

### Plasmids

pcDNA3.1-MondoA-V5, pcDNA3-Mlx-FLAG, and LXSH-MondoA as well as TXNIP promoter luciferase reporter plasmids (wild type and ChoRE mutant) have been described [12, 16]. pcDNA3-Mit-ATEAM (pcDNA3-mitAT1.03) was a gift of Hiroyuki Noji, Rikkyo University [17]. pLKO.1-shScrm and pLKO.1-shTXNIP were obtained from Sigma Aldrich. Standard molecular cloning techniques were used to generate pLVX-TetOne-Puro-MYC(T58A). The pLVX-TetOne-Puro vector was obtained from Clontech Laboratories. Transfections were performed using Lipofectamine 2000 (Thermo Fisher) or Lipofectamine 3000 (Thermo Fisher).

### Protein synthesis inhibitor treatments

Growth media was replaced with glucose-free DMEM with penicillin/streptomycin and 10% FBS for 6 h. Media was then replaced with glucose-containing DMEM with penicillin/streptomycin, 10% FBS, and translation inhibitors for 16 h. Unless otherwise indicated, the protein synthesis inhibitors were added at the following concentrations: cycloheximide (CHX) (Sigma Aldrich), 50 μg/ml; emetine (Sigma Aldrich), 100 μg/ml; puromycin (Puro) (Sigma Aldrich), 100 μg/ml; and rocaglamide A (Santa Cruz), 25–100 nM. Dialyzed FBS was prepared by dialysis 3 times against 40-fold excess water to remove small molecules.

### Quantitative real-time PCR

Total RNA was extracted using the RNAeasy Kit (Qiagen). cDNA was synthesized from 0.1 to 1 μg RNA using GoScript reverse transcription kit (Promega). qPCR was performed using OneTaq Hot Start DNA Polymerase [18], SYBR/ROX Combo PCR DNA Fluorescence Dye (Thermo Fisher), and dNTPs (Thermo Fisher). The ΔΔCt method with normalization to actin levels was used to analyze the data. Three biological replicates were used to determine mRNA levels and calculate significance. Three technical replicates were performed for every biological sample. TXNIP primers: forward—TGACTTTGGCCTACAGTGGG and reverse—TTGCGCTTCTCCAGATACTGC; Actin primers: forward—TCCATCATGAAGTGTGACGT and reverse—TACTCCTGCTTGCTGATCCAC.

### Immunofluorescence

Cells were transfected with plasmids containing MondoA-V5 and FLAG-Mlx using Lipofectamine 2000 (Thermo Fisher). Following protein synthesis inhibitor treatment, cells were fixed on glass coverslips using ice-cold 100% methanol for 15 min and stained using standard immunofluorescence procedures. Mouse anti-V5 (Thermo Fisher) antibody was used at 1:2000; rabbit anti-FLAG (Cell Signaling) antibody was used at 1:2000.

### Metabolomics

Gas chromatograph-mass spectrometry (GC-MS) was used to determine metabolite levels as described previously [13]. Metabolites were harvested from cells using 90% methanol and analyzed over a 30 m Phenomex ZB5-5 MSi column. Data was analyzed using the MassLynx 4.1 software (Waters). Six biological replicates were used for each treatment group. Peak areas, normalized for total ion current for each sample, for individual metabolites were determined and used to calculate fold change after treatment with CHX. All analyzed metabolites are presented in Supplemental Figure 1.

### Chromatin immunoprecipitation

MondoA-V5 was transfected into HeLa cells. Chromatin was cross-linked and sheared as described [19]. Chromatin was incubated overnight with anti-V5 antibody (Thermo Fisher) or mouse IgG (Sigma Aldrich). M-280 sheep anti-mouse Dynabeads (Thermo Fisher) were used to capture and purify immunocomplexes. DNA was purified using a QIAquick PCR Purification Kit (Qiagen) and analyzed using quantitative PCR as described above. Primers were previously described [20].

### Promoter activity assay

TXNIP promoter luciferase assays were performed as described previously [21]. Briefly, cells were transfected with a TXNIP promoter luciferase construct and a CMV-driven β-galactosidase construct. Following treatments, luciferase and β-galactosidase activities were determined according to manufacturer’s recommendations (Promega, Tropix). Luciferase activity was normalized to β-galactosidase to control for differences in transfection efficiency.

### Fluorescence resonance energy transfer (FRET)

Widefield microscopy was used to perform live cell imaging on cells expressing Mit-ATEAM as described previously [13]. Briefly, cells transiently transfected with pcDNA3-Mit-ATEAM. Live imaging was conducted using a Nikon A1R with a × 40 lens. Images were captured every hour for 6 h using the following channels: yellow fluorescent protein (YFP) (excitation 488 nm, emission 525 nm), cyan fluorescent protein (CFP) (excitation 405 nm, emission 480 nm), and FRET (excitation 405 nm, emission 525 nm). The YFP channel was used to designate mitochondria area. The ratio of CFP intensity to FRET intensity was used to determine relative mitochondrial ATP levels.

### Immunoblotting

Immunoblotting was performed as described previously [19]. Primary antibodies were used at a dilution of 1:1000 anti-MLXIP/MondoA (Proteintech, 13614-1-AP), 1:2000 anti-VDUP1/TXNIP (MBL, K0205-3), 1:15,000 anti-Tubulin (Molecular Probes, 236-10501), and 1:1000 anti-EIF4E (BioLegend, 693002). Secondary antibodies were used at a dilution of 1:5000 anti-rabbit-HRP (GE Life Sciences, NA-934) or 1:15,000 anti-mouse-HRP (GE Life Sciences, NA-931).

### Cell viability assay

Crystal violet staining was used to determine relative cell viability/proliferation. Cells were stained/fixed using a mixture of 0.05% crystal violet, 1% formaldehyde, 1% methanol, 137 mM NaCl, 2.7 mM KCl, 10 mM Na_2_HPO_4_, and 1.8 mM KH_2_PO_4_. Following 1 h in the staining/fixing solution, cells were washed with water until all excess stain was removed, and the plates were dried at room temperature. Crystal violet was extracted from the cells using 1% SDS and absorbance at 590 nm was used as a relative measure of total cell numbers.

### mRNA sequencing and analysis

mRNA sequencing was performed as described previously [13]. RNA was harvested using a Quick RNA Miniprep kit (Qiagen), and cDNA libraries were constructed using a stranded mRNA-seq Kit with mRNA Capture Beads (Kapa). The library was sequenced using an Illumina HiSeq 2500. Sequencing was performed by Huntsman Cancer Institute’s High Throughput Genomics Core. Reads were aligned to the human genome using STAR. DESeq2 was used to determine differential expression of genes. To determine regulated pathways, we conducted (1) overrepresentation analysis using ConsensusPathDB [22] and (2) gene set enrichment analysis and leading-edge analysis (Broad Institute) [23, 24].

### Patient-derived xenograft organoids

Patient-derived xenograft (PDX) tumors were harvested, processed into organoids (PDxOs) [25–27], and cultured exclusively in a 3D matrigel environment (Corning, growth factor reduced). Fully mature organoids, > 50 μm in diameter, were seeded at a density of 50–100 organoids/well in 5% matrigel, into 384-well tissue cultures plates—coated with matrigel to prevent adhesion. Twenty-four hours after seeding, PDxOs were treated with serial dilutions of RocA. We assayed cell viability prior to treatment and after 4 days of treatment with CellTiter-Glo 3D (Promega). Response was determined from technical quadruplicates over three biological replicates.

### Statistical methods

Data represents the mean ± S.D. for five biological replicates for metabolomics experiments and three biological replicates for all other experiments including RNA-seq. Analysis of variance (ANOVA) was performed to determine significance.

## Results

### Translation inhibition drives TXNIP expression

**Fig. 1:**
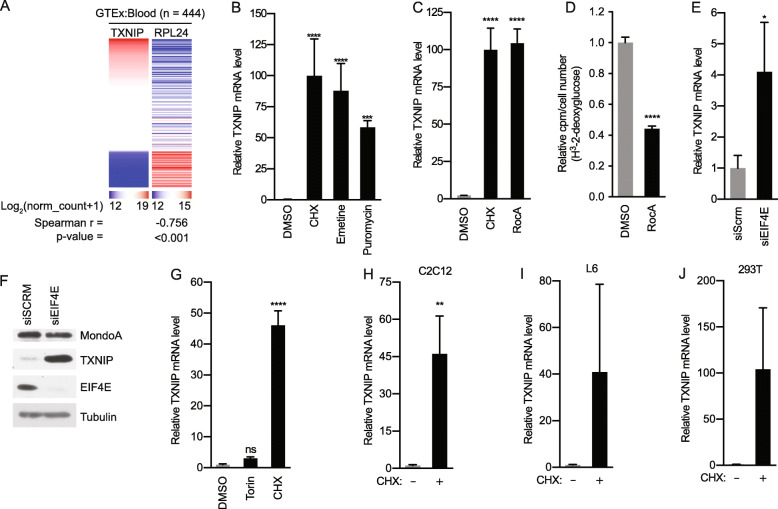
TXNIP expression is suppressed by translation. **a** Heatmaps showing TXNIP mRNA relative to ribosomal protein L24 in the genotype-tissue expression project (GTEx) database. Spearman correlation statistics are reported. **b**, **c** TXNIP mRNA levels in HeLa cells following 16-h treatments with the translation elongation inhibitors CHX (50 μg/mL), emetine (50 μg/mL), and puromycin (100 μg/mL) or the translation initiation inhibitor Rocaglamide A (RocA, 100 nM). **d** Relative rate of ^3^H-2-deoxyglucose uptake in HeLa cells following 16-h treatments with RocA or vehicle. **e**, **f** TXNIP mRNA and protein levels of the indicated proteins in HeLa cells transfected with a pool of four siRNAs against EIF4E or a pool of four scrambled siRNA controls. **g** TXNIP mRNA levels in HeLa cells following 16-h treatments with CHX or Torin (250 nM). TXNIP mRNA levels following 16-h CHX treatments of **h** C2C12 mouse myoblasts, **i** L6 rat myoblasts, and **j** 293T embryonic kidney cells. TXNIP mRNA levels were determined using RT-qPCR

To determine whether TXNIP expression is generally correlated with protein synthesis, we investigated how the expression of a known translation regulator correlates with TXNIP expression. Using the Gene-tissue Expression Database (GTEx) and examining expression in the blood, we identified a strong negative correlation between TXNIP and ribosomal protein L24 (RPL24), which has been shown to correlate well with global changes in protein synthesis in lymphocytes [28, 29]. This finding supports the hypothesis that high translation rates suppress TXNIP expression (Fig. 1a).

We next determined whether compounds that block translation at different steps regulate *TXNIP* expression. We found that treatment of Hela cells with three translation elongation inhibitors, cycloheximide (CHX), emetine, and puromycin (Puro), increased *TXNIP* expression dramatically (Fig. 1b). Likewise, the translation initiation inhibitor RocA [30] induced *TXNIP* expression comparably to CHX (Fig. 1c). As expected [6], TXNIP induction by RocA was accompanied by a decrease in glucose uptake (Fig. 1d). siRNA-mediated knockdown of translation initiation factor EIF4E also increased TXNIP expression (Fig. 1e, f), confirming our findings with the pharmacological inhibitors. It is counter-intuitive that TXNIP protein would accumulate following knockdown of EIF4E; however, TXNIP undergoes both cap-dependent and internal ribosome entry site (IRES)-dependent translation [31]. Therefore, we speculate that IRES-dependent translation accounts for the increase in TXNIP protein levels following EIF4E knockdown. Our previous studies demonstrated that mammalian target of rapamycin complex 1 (mTORC1) suppresses MondoA transcriptional activity and TXNIP expression by competing for its obligate transcriptional partner Mlx [19]. Consistent with our previous findings, the mTORC1 inhibitor Torin increased TXNIP expression, but this increase was much more modest than that observed with CHX (Fig. 1g). This finding suggests that broad translation inhibitors like RocA and CHX increase TXNIP expression by a different mechanism than does Torin, and their action is largely independent of mTORC1. Finally, CHX increased *TXNIP* expression in C2C12 and L6 myoblasts and HEK293T embryonic kidney cells (Fig. 1h–j), suggesting that protein synthesis inhibitors generally increase TXNIP expression. Together, these findings suggest that *TXNIP* expression, and consequently glucose uptake, is tightly linked to translation rate.

### Protein synthesis inhibitors drive MondoA transcriptional activity

**Fig. 2:**
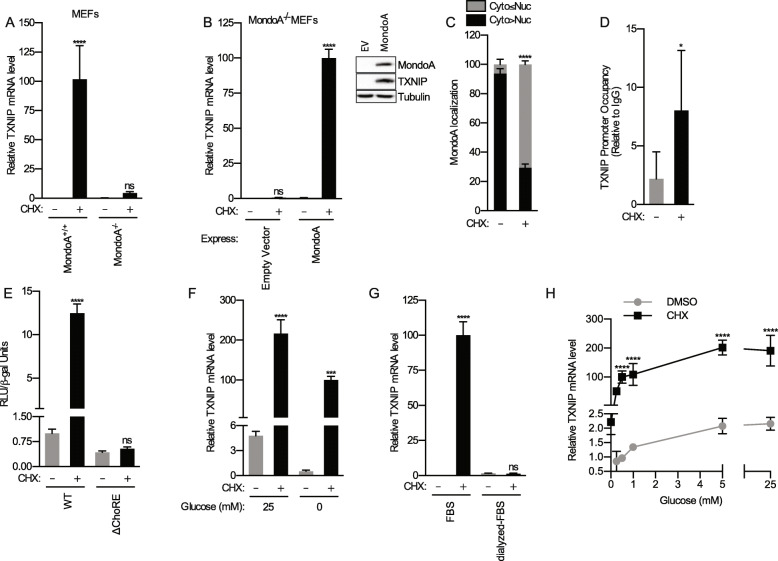
Protein synthesis inhibition drives MondoA transcriptional activity. TXNIP mRNA levels following 16-h CHX treatments of **a** MondoA+/+ and MondoA−/− MEFs, and **b** MondoA−/− MEFs expressing empty vector or MondoA. **c** Immunofluorescence was used to assess the subcellular localization of MondoA in HeLa cells treated with CHX for 16 h. Cells were scored for localization of MondoA (cytoplasmic > nuclear or cytoplasmic ≤ nuclear). **d** Chromatin immunoprecipitation was used to determine the enrichment of MondoA on the TXNIP promoter in HeLa cells treated with CHX for 16 h. **e** HeLa cells transfected with the indicated reporter luciferase constructs were treated with CHX for 16 h. The ChoREmut TXNIP promoter lacks the double CACGAG carbohydrate responsive element located directly upstream of luciferase. The media was replaced with regular medium for 1 h to wash out CHX, allowing translation of accumulated luciferase mRNA. **f** To ensure that TXNIP levels were at a minimum, HeLa cells were starved of glucose for 6 h prior to treatment with CHX. We then measured TXNIP mRNA levels in cells growing in DMEM +10% FBS or glucose-free DMEM +10% FBS following 16-h CHX treatments. **g** TXNIP mRNA levels in HeLa cells growing in glucose-free DMEM +10% FBS or in glucose-free DMEM +10% dialyzed FBS following 16-h treatments. **h** TXNIP mRNA levels in HeLa cells growing in glucose-free DMEM +10% dialyzed FBS with the indicated amount of glucose following 16-h treatments with CHX. TXNIP mRNA levels were determined using RT-qPCR

We next evaluated the involvement of MondoA in *TXNIP* induction in response to protein synthesis inhibition. CHX treatment increased *TXNIP* expression, in wildtype but not in MondoA^−/−^ mouse embryonic fibroblasts (MEFs) (Fig. 2a). Ectopic expression of MondoA in MondoA^−/−^ MEFs rescued *TXNIP* induction (Fig. 2b). We tested whether CHX increased MondoA transcriptional activity using several approaches. First, the nuclear localization of MondoA and the amount of MondoA on the TXNIP promoter increased following CHX treatment (Fig. 2c, d). Second, CHX increased the expression from a TXNIP luciferase reporter construct in a manner that was strongly dependent on an intact CACGAG ChoRE about 80 bp upstream of the transcription start site (Fig. 2e). Together these data demonstrate that CHX, and likely other protein synthesis inhibitors, drives MondoA nuclear accumulation, promoter binding, and transcriptional activity.

Because MondoA transcriptional activity is strictly dependent on glucose [10, 11], we next determined the requirement for glucose in CHX-driven TXNIP expression. HeLa cells were treated with CHX in DMEM or in glucose-free DMEM. Surprisingly, *TXNIP* was induced in both media conditions (Fig. 2f), suggesting that CHX might induce MondoA transcriptional activity independent of glucose. An alternate possibility is that fetal bovine serum (FBS) contains sufficient glucose (~ 5 mM) such that when present in culture medium at 10% the resulting concentration of glucose (~ 0.5 mM) can support MondoA transcriptional activity. To test this hypothesis, we dialyzed FBS to remove small molecules including glucose and then treated cells with CHX in glucose-free DMEM + 10% dialyzed FBS. CHX did not increase *TXNIP* expression in medium containing dialyzed serum; however, adding glucose back to the medium that contained dialyzed serum rescued TXNIP induction (Fig. 2g, h). CHX increased TXNIP expression at all glucose levels tested, and surprisingly decreased the threshold of glucose required for *TXNIP* induction ~ 5-fold (Fig. 2h). Further, RocA showed glucose-dependent changes in *TXNIP* expression (Supplemental Figure 1A). Thus, glucose is strictly required for CHX to increase MondoA transcriptional activity and also sensitizes MondoA transcriptional activity to lower glucose levels.

### Protein synthesis inhibition drives G6P production

**Fig. 3:**
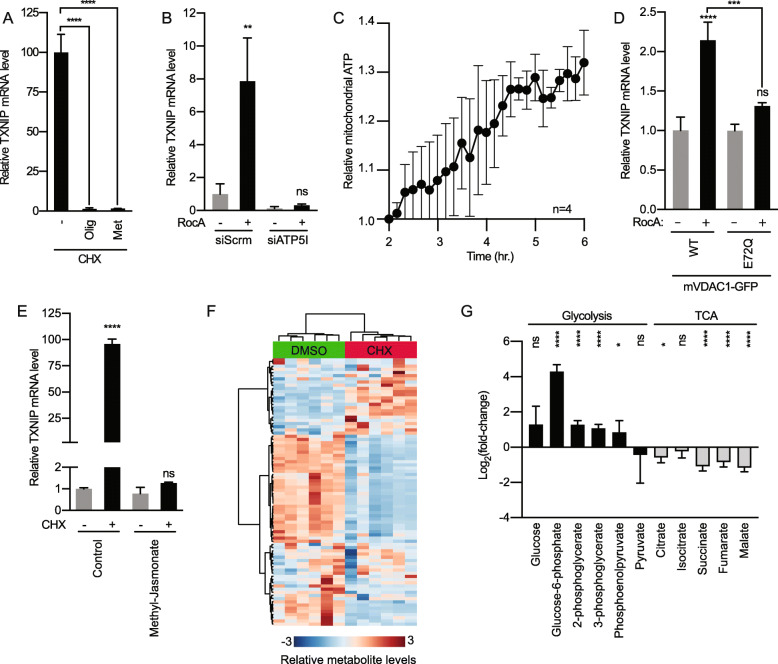
Protein synthesis inhibition drives G6P synthesis. **a** TXNIP mRNA levels in HeLa cells treated with CHX and the electron transport chain inhibitor metformin (Met, 5 mM) or oligomycin (Olig, 1 μM) for 16 h. **b** TXNIP mRNA levels following a 16-h RocA treatment of HeLa cells transfected with pool of siRNA specific for ATP5I (siATP5I) or a pool of scrambled control siRNAs (siSCRM). **c** A mitochondrial-targeted ATP FRET biosensor (mitATEAM) was used to determine relative mtATP levels in HeLa cells treated with the protein synthesis inhibitor RocA for up to 6 h. Relative mtATP was determined as the ratio of FRET to CFP intensities. **d** TXNIP mRNA levels following a 16-h RocA treatment (100 nM) of HeLa cells expressing wildtype mouse VDAC1 (mVDAC1) or VDAC1(E72Q), which cannot bind Hexokinase II. **e** TXNIP mRNA levels in HeLa cells treated for 16 h with CHX or methyl-jasmonate (3 mM). **f** Heatmap showing relative metabolite levels from HeLa cells treated with CHX for 16 h. Metabolite levels were assessed through GC-MS. **f** Log_2_(fold-change) of glycolytic and TCA cycle intermediates from HeLa cells treated with CHX for 16 h, relative to control DMSO treatment. TXNIP mRNA levels were determined using RT-qPCR

We next investigated how protein synthesis inhibitors increase MondoA transcriptional activity. We focused on a potential role for mitochondrial function and mtATP for three reasons: (1) protein translation is the most ATP-consuming biosynthetic reaction, (2) MondoA transcriptional activity depends on mtATP [13], (3) higher mtATP levels may sensitize MondoA transcriptional activity and TXNIP expression to lower levels of glucose by increasing levels of G6P [13]. Consistent with a requirement for functional electron transport, inhibition of complex I with metformin completely abrogated TXNIP induction by CHX (Fig. 3a). Likewise, and consistent with a requirement for mtATP, blocking the activity of ATP synthase (complex V) with oligomycin also robustly inhibited TXNIP expression (Fig. 3a). To test the requirement of ATP synthesis further, we used siRNA to deplete ATP5I, which is an essential component of ATP synthase: our previous work established that ATP5I knockdown in HeLa cells blocks the production of mtATP [13]. In this experimental context, ATP5I knockdown reduced background TXNIP expression and completely suppressed its induction by RocA (Fig. 3b). We next determined how protein synthesis inhibition affects mtATP. We expressed a mitochondrial-targeted ATP FRET-biosensor (mitATEAM) in HeLa cells and used live cell imaging to quantify fluorescence [13, 17]. Inhibiting protein synthesis by RocA leads to increased FRET signal indicating accumulation of ATP in the mitochondria (Fig. 3c and Supplemental Figure 1B). These findings suggest a requirement for mtATP synthesis in driving TXNIP expression in response to protein synthesis inhibition.

Low pH medium increases MondoA transcriptional activity by increasing mtATP levels [13]. In that work, we established that mtATP exits the mitochondrial matrix via a channel comprised of the adenine nucleotide transporter (ANT) and the voltage-dependent anion channel (VDAC), where it is used as substrate for VDAC-bound hexokinase II (HKII). Mitochondria-bound HKII then transfers a phosphate to cytoplasmic glucose to generate G6P resulting in a stimulation of MondoA transcriptional activity. We tested whether RocA induces TXNIP expression through a similar mechanism in three ways. First, expression of VDAC1(E72Q), which cannot interact with HKII and prevents HKII from interacting with mitochondria [13, 32], blocked the increase in TXNIP expression following RocA treatment (Fig. 3d). By contrast, wildtype VDAC increased TXNIP expression in the presence of RocA. Second, methyl-jasmonate, which removes HKII from the outer membrane of mitochondria [33], blocked RocA induction of TXNIP (Fig. 3e). Third, CHX leads to a dramatic reprogramming of metabolism, including significant changes in the levels of glycolytic and TCA cycle intermediates (Fig. 3f, g and Supplemental Table 1). In particular, G6P levels increased more than 20-fold following CHX treatment (Fig. 3g). Together, these data are consistent with the model that protein synthesis inhibitors increase mtATP, which is subsequently exported from the mitochondrial matrix through the ANT/VDAC channel, ultimately increasing G6P levels to drive MondoA transcriptional activity.

### MondoA and TXNIP are required for the cytotoxic effects of RocA

**Fig. 4:**
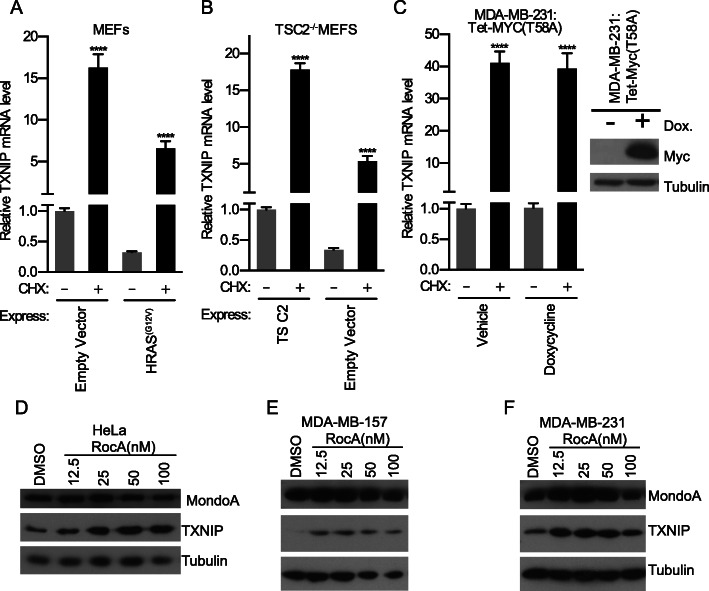
Protein synthesis inhibition drives TXNIP expression independent of oncogenic burden. TXNIP mRNA levels following a 16-h CHX treatment of **a** wildtype or HRAS(G12V)-expressing murine embryonic fibroblasts (MEFs), **b** TSC2−/− MEFs expressing empty vector or human-TSC2, and **c** MDA-MB-231 expressing tet-inducible MYC(T58A) with or without doxycyline. Immunoblots showing TXNIP, MondoA, and tubulin protein levels following 16-h RocA treatment of **d** HeLa cells, **e** MDA-MB-157 cells, and **f** MDA-MB-231 cells. TXNIP mRNA levels were determined using RT-qPCR

Because protein synthesis inhibitors are emerging as potential cancer therapeutics [4–7], we tested whether blocking protein synthesis induced TXNIP expression in cell lines with different oncogenic lesions. CHX induced TXNIP in MEFs and in MEFs that expressed an activated allele of HRAS (Fig. 4a) [34]. Further, TXNIP was induced by CHX-treatment in MEFs that lack the TSC2 tumor suppressor and in MDA-MDA-231 cells, which is a triple negative breast cancer (TNBC) cell line that harbors an inactivating mutation in TP53 and activating mutations in KRAS and BRAF (Fig. 4b, c). Further, induction of c-Myc(T58A), which is a stabilized allele of c-Myc, did not block TXNIP induction in MDA-MB-231 cells (Fig. 4c). RocA also increased TXNIP protein levels in HeLa cells, MDA-MB-157 cells, which is also a TNBC cell line, and in MBA-MB-231 cells (Fig. 4d, e, f). Together these data demonstrate that RocA can induce TXNIP expression in a variety of cell lines, and its action appears relatively independent of oncogenic burden.

**Fig. 5:**
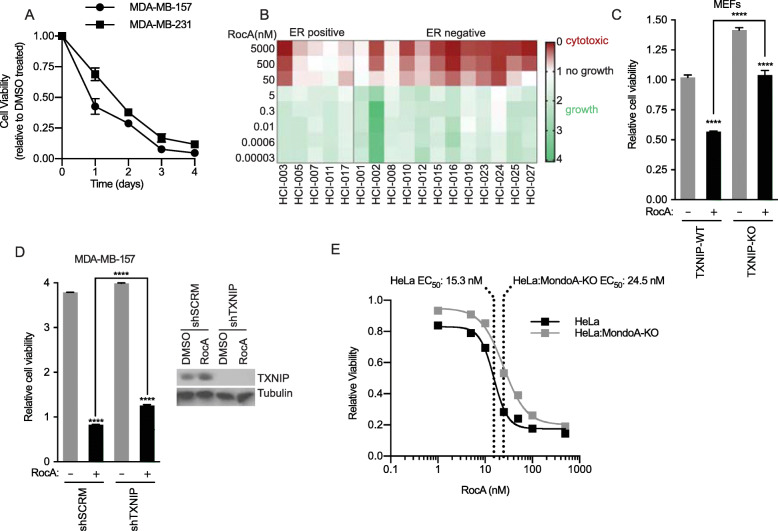
Cytotoxicity elicited by protein synthesis inhibitors requires TXNIP. **a** Relative cell viability over the indicated time course of MDA-MB-157 and MDA-MB-231 cells in the presence of RocA (100 nM) was assessed by crystal violet staining. **b** Viability of patient-derived xenograft organoids (PDxOs) following treatment with RocA at various concentrations. PDxOs are separated into ER+ and ER− groups. **c** TXNIP+/+ or TXNIP−/− MEFS were treated with RocA for 2 days; then, cell viability was analyzed using crystal violet staining. **d** MDA-MB-157 cells expressing scrambled shRNA (shScrm) or shTXNIP were treated with 100 nM RocA for 2 days; then, cell viability was analyzed using crystal violet staining. **e** We previously characterized HeLa cells in which MondoA was knocked out by CRISPR/Cas9 [13]. Cells were treated with RocA for 2 days, and then, cell viability was analyzed using crystal violet staining

The growth inhibitory effect of RocA has been tested primarily on multiple myeloma cell lines [6, 35]. Consistent with a potential broad effect of RocA on cell growth, treatment of MDA-MB-157 and MDA-MB-231 breast cancer cells with 100 nM RocA resulted in a time-dependent reduction in cell viability such that virtually all the cells were dead after 4 days of treatment (Fig. 5a). We expanded this analysis to 17 organoid cultures derived from breast cancer patients treated at Huntsman Cancer Institute. As with the cell lines, these patient-derived xenograft organoids (PDxOs), showed sensitivity to RocA. Ten of 12 estrogen receptor negative (ER−) models were sensitive to RocA, with consistently strong cytotoxicity around 50 nM (Fig. 5b). Most of the estrogen receptor positive (ER+) models, with the exception of HCI-011, were also sensitive to RocA, but sensitivity was attenuated compared to the ER− models: HCI-003 was highly sensitive to RocA like the majority of the ER− models. Thus, RocA is broadly cytotoxic to breast cancer cells and appears to show preferential killing of cells from ER− breast cancers.

We next determined whether MondoA or TXNIP was required to mediate the cytotoxic effects of RocA. TXNIP-knockout MEFs were less susceptible to RocA than wildtype MEFs (Fig. 5c), consistent with the notion that TXNIP is a RocA effector. Likewise, TXNIP knockdown in MDA-MB-157 cells also partially blocked the cytotoxic effects of RocA (Fig. 5d). Finally, we used CRISPR-Cas9 editing to generate HeLa cells that lack MondoA (HeLa:MKO) and conducted a RocA dose response experiment. While MondoA-knockout had no effect on cell proliferation in the absence of RocA (Supplemental Figure 1C), we observed that MondoA loss attenuated the cytotoxic effects of RocA and increased the IC50 of RocA from ~ 15 to ~ 25 nM (Fig. 5e). Together these data suggest induction of MondoA transcriptional activity, and the subsequent induction of TXNIP is required for the full cytotoxic effects of RocA. However, the effect of MondoA and TXNIP loss on RocA cytotoxicity, while significant, is subtle suggesting that other pathways must also contribute.

### The role of MondoA in the transcriptional response to RocA

**Fig. 6:**
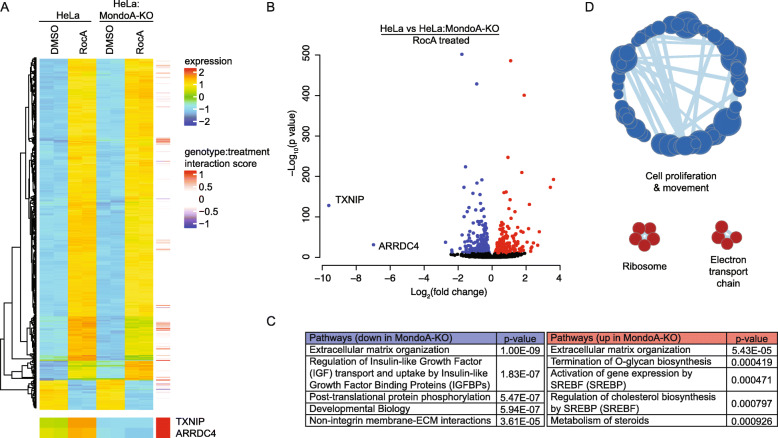
The MondoA-dependent transcriptional response to translation inhibition. mRNA sequencing was used to determine gene expression changes in HeLa and HeLa:MondoA-KO cells following 4-h treatments with 100 nM RocA. **a** Heatmap depicting the top 500 genes regulated by RocA treatment. Regression analysis using DESeq2 was performed to generate a genotype:treatment interaction scores. **b** A volcano plot showing log2(fold-change) of HeLa cells treated with RocA compared to HeLa:MondoA-KO cells treated with RocA. Genes with an adjusted *p* value ≤ 1E−10 are indicated in blue (downregulated) and red (upregulated). **c** Overrepresentation analysis was used to determine pathways that are dysregulated in HeLa cells treated with RocA compared to HeLa:MondoA-KO cells treated with RocA. **d** Gene set enrichment analysis and leading-edge analysis was performed using all gene sets in the Molecular Signature Database (Broad Institute). HeLa cells treated with RocA were compared to HeLa:MondoA-KO cells treated with RocA. Nodes that contain at least 4 gene sets are shown

To understand the contribution of MondoA to the RocA-dependent transcriptional response, we conducted mRNA-sequencing on RNA prepared from wildtype HeLa or HeLa:MKO cells that had been treated for with 100 nM RocA for 4 h. Using a 2-fold expression change cutoff and a *p* value of ≤ 0.01, we identified 1241 genes that were differentially regulated by RocA. Of these, 224 genes were not differentially regulated in the absence of MondoA. This finding suggests that approximately 20% (224/1241) of the RocA-driven transcriptome requires MondoA (Fig. 6a): both up- and downregulated genes were MondoA-dependent. We next used regression analysis to look for genes that were affected by RocA treatment and genotype. As expected, TXNIP was highly induced by RocA, and its expression was highly dependent on MondoA (Fig. 6a, b). Induction of the TXNIP paralog arrestin domain containing 4 (ARRDC4) by RocA was less robust but was also highly MondoA-dependent (Fig. 6a, b). Pathways downregulated following RocA treatment of MondoA knockout cells included extracellular matrix organization and a number of signaling-related pathways (Fig. 6c) [22]. Pathways upregulated following RocA treatment of MondoA knockout cells also included extracellular matrix organization and several pathways involved in sterol biosynthesis. Finally, we conducted gene set enrichment analysis on the differentially regulated genes in HeLa and HeLa:MondoA-KO cells treated with RocA using 13445 pathways in the Molecular Signatures Database [23, 24]. We identified 1033 gene sets that were enriched with a nominal *p* value of ≤ 0.01. Leading-edge analysis showed that pathways associated with cell proliferation and cell movement were upregulated, and electron transport and ribosome-related pathways were downregulated in RocA-treated HeLa:MKO cells (Fig. 6d). Together, these data suggest that MondoA is required for the cellular transcriptional response to RocA treatment and may contribute to migratory and growth phenotypes driven by protein synthesis inhibitors.

## Discussion

Translation rate is positively linked to the availability of progrowth signals and the availability of nutrients and charged amino acids [36–38]. A previous report showed that RocA induced TXNIP [6], which correlated with a downregulation of glucose uptake and a blockade of cell growth. Here, we provide a mechanistic framework for this observation, showing that multiple protein synthesis inhibitors, including RocA, drive TXNIP expression by increasing MondoA transcriptional activity. These findings link translation rate to glucose uptake through regulation of MondoA transcriptional activity. We show that protein synthesis inhibitors induce TXNIP expression in a number of different cell lines, apparently independent of oncogenic burden. This finding complements earlier studies showing that a compound related to RocA induces TXNIP expression in a number of cancer cell lines representing a spectrum of malignancies [6].

Cancer cells must coordinate the use and the availability of nutrients to support growth and division. TXNIP is a potent negative regulator of glucose uptake; in fact, its loss or downregulation is sufficient to increase glucose uptake [19, 39], suggesting that low TXNIP levels may be a common route to aerobic glycolysis common in cancer. Consistent with this hypothesis, TXNIP levels are generally lower in tumors compared to normal adjacent tissues [8], and a number of pro-growth/oncogenic pathways suppress TXNIP expression by a variety of mechanisms [19, 20, 34, 40–42]. Together these data suggest that the high demand for ATP driven by translation may result in a reduction of G6P, reduced MondoA transcriptional activity, and low TXNIP expression. Low TXNIP levels increase glucose uptake to help sustain ATP production through glycolysis and potentially replenish stores of glucose-derived amino acids.

TXNIP expression is increased by serum starvation and via inhibition of growth factor signaling pathways [40, 43]. These treatments also restrict protein translation, raising the possibility that they increase TXNIP expression by altering mATP and/or G6P pools. However, the magnitude of TXNIP induction driven by serum starvation or inhibition of growth factor signaling is much less than the increase in TXNIP levels we observe following CHX or RocA treatment. Similarly, we have previously shown that mTORC1 negatively regulates MondoA transcriptional activity and TXNIP expression by binding to MondoA’s obligate partner Mlx [19]. Again, the effect of mTORC1 on MondoA activity is relatively subtle compared to effect of CHX and RocA (Fig. 2 c and g). Together these data suggest that MondoA transcriptional activity is regulated in a relatively tight window under physiological growth conditions, but the supraphysiological levels of mATP and G6P driven by broad inhibition of protein synthesis increases MondoA activity much more potently. Nonetheless, we implicate G6P in activating MondoA transcriptional activity under both physiological conditions and following protein synthesis inhibition. Furthermore, CHX reduces the threshold of glucose needed to drive MondoA transcriptional activity about 5-fold (Fig. 2f). Together these data suggest that inhibition of protein synthesis amplifies the normal regulatory mechanisms that control MondoA transcriptional activity, rather than controlling MondoA activity via an alternate de novo mechanism.

We and others showed previously that MondoA is a critical regulator of glucose-induced transcription [10], which is triggered by G6P. We reported previously that low pH (~ 6.7) triggers MondoA transcriptional activity and TXNIP expression [44]. Our recent report demonstrated that low pH triggers TXNIP expression by increasing mtATP production [13]. Under low pH conditions mtATP is exported from the mitochondrial matrix, encountering hexokinase II at the outer mitochondrial membrane and generating G6P from cytoplasmic glucose to trigger MondoA transcriptional activity. We show here that inhibition of protein synthesis drives increases MondoA transcriptional activity by a similar mechanism. RocA’s induction of MondoA transcriptional activity depends on mtATP synthesis and the interaction of HKII with the outer mitochondrial membrane. Further, our metabolomics experiment showed that CHX treatment results in a dramatic increase in G6P and several other glycolytic intermediates, whereas most TCA intermediates are reduced under these conditions. Together these data suggest that blocking protein synthesis drives MondoA transcriptional activity by increasing mtATP levels, followed by export of mtATP from the mitochondrial matrix and the subsequent increase in G6P: increased G6P triggers MondoA transcriptional activity. It seems most likely that protein synthesis blockade increases mtATP levels by reducing the cytoplasmic demand for ATP. We are currently exploring this and other possibilities.

We showed that both MondoA and TXNIP are partially required for the growth suppressive activity of RocA, suggesting that the increase in MondoA activity and in TXNIP expression are just not correlated with protein synthesis inhibition, but may be critical for the full therapeutic response to protein synthesis blockade. Our previous work demonstrated that a number of progrowth pathways inhibit MondoA transcriptional activity and TXNIP expression [19, 34, 40, 41], suggesting a potential limitation of protein synthesis inhibitors as cancer therapeutics. However, we show that RocA induced MondoA activity and TXNIP expression in several cell lines independent of oncogenic burden. While our current experiments focus on TXNIP induction by RocA, our previous work demonstrated that a slightly acidic pH of ~ 6.7 drives a gene signature that correlates with good clinical prognosis in breast cancer, and TXNIP is a component of that signature [44]. These findings argue that identifying or developing more specific TXNIP inducers may have therapeutic utility.

In addition to its inhibitory effects on eIF4A, RocA also been shown to disrupt Ras-Raf-MEK signaling. This occurs through the direct binding of prohibitins (PHB1 and PHB2) and their sequestration in the cytosol, which prevents Raf localization to the plasma membrane and its activation by Ras [45]. Given our previous findings that Ras-Raf signaling prevents MondoA transcriptional activity and TXNIP expression [34, 40, 41], it is possible that RocA-driven inhibition of Ras-Raf-MEK signaling also contributes to the increase in MondoA transcriptional activity we observe with RocA treatment.

Finally, MondoA is required for the adaptive transcriptional program driven by RocA and accounts for ~ 20% of the RocA-induced changes in gene expression. Consistent with our recent demonstration that TXNIP and its paralog ARRDC4 are the principal direct MondoA targets in response to acidosis [12, 13, 16], their expression is also highly MondoA- and RocA-dependent in these experiments. Leading edge analysis indicates that multiple pathways involved in ribosome function and electron transport chain activity are downregulated in response to RocA, supporting the possibility that translation rate is coupled to mitochondrial function and mtATP levels. Conversely, multiple cell proliferation and migration pathways are upregulated in response to RocA, perhaps reflecting increased mtATP levels. Further experiments will be necessary to fully understand the biological impact the MondoA-dependent changes in gene expression following protein synthesis inhibition.

## Conclusions

Here we show that protein synthesis inhibitors, including specific inhibitors of translation initiation, increase MondoA transcriptional activity resulting in elevated TXNIP expression. Given that TXNIP is a potent negative regulator of glucose uptake, our results suggest coordination between translation rate and glucose availability. Mechanistically, inhibition of protein translation increases mtATP, ultimately increasing G6P levels to trigger MondoA transcriptional activity. The MondoA-TXNIP axis is required for the full cytotoxic effect of RocA, suggesting that negative regulators of MondoA transcriptional activity may limit the efficacy of translational inhibitors in clinical settings. Finally, patient-derived organoid models of ER− breast cancers are particularly sensitive to RocA, potentially providing a new therapeutic option against this aggressive and difficulty-to-treat breast cancer subtype.

## Supplementary Information

**Additional file 1: Figure S1.** (A) TXNIP mRNA levels following 16-hour CHX or RocA treatments of HeLa cells in glucose-free or high-glucose media, each with 10% FBS. TXNIP mRNA levels were determined using RT-qPCR. (B) Widefield confocal images of HeLa cells expressing mit-ATEAM and treated with RocA for indicated times. The coloring is representative of the ratio of FRET to CFP, where orange/yellow corresponds to higher levels of mtATP. (C) Relative proliferation rate of HeLa and HeLa:MondoA-KO cells over the course of 4 days were analyzed by crystal violet (*n* = 3 for each cell line).

**Additional file 2: Table S1.**

## Data Availability

RNA-seq data is available at GEO under the accession number GSE153499. The metabolomics dataset is available in Supplemental Table 1. All of the remaining data generated or analyzed during this study are included in this published article and its supplementary information files.

## Abbreviations

ATP: Adenosine triphosphate
TXNIP: Thioredoxin-interacting protein
RocA: Rocaglamide A
G6P: Glucose 6-phosphate
PDxO: Patient-derived xenograft organoid
mtATP: Mitochondrial ATP
VDAC: Voltage-dependent anion channel
HK: Hexokinase
TNBC: Triple negative breast cancer
ER−: Estrogen receptor negative
ROS: Reactive oxygen species
OMM: Outer mitochondrial membrane
ChoRE: Carbohydrate response element
DMEM: Dulbecco’s Minimal Essential Media
FBS: Fetal bovine serum
CHX: Cycloheximide
Puro: Puromycin
GC-MS: Gas chromatograph-mass spectrometry
FRET: Fluorescence resonance energy transfer
YFP: Yellow fluorescent protein
CFP: Cyan fluorescent protein
PDX: Patient-derived xenograft
ANOVA: Analysis of variance
GTEx: Gene-tissue Expression Database
RPL24: Ribosomal protein L24
IRES: Internal ribosome entry site
mTORC1: Mammalian target of rapamycin complex 1
MEFs: Mouse embryonic fibroblasts
ANT: Adenine nucleotide transporter
ER+: Estrogen receptor positive
ARRDC4: Arrestin domain containing 4
Met: Metformin
Olig: Oligomycin
