# Supplementary material for: Protein synthesis inhibitors stimulate MondoA transcriptional activity by driving an accumulation of glucose 6-phosphate
Source: Cancer Metab. 2020 Dec 4;8:27. doi: 10.1186/s40170-020-00233-6 (PMC7718662; doi:10.1186/s40170-020-00233-6)
Supplement: Supplementary file 2 — Additional file 2: Table S1. [file 40170_2020_233_MOESM2_ESM.pdf]

	CHX 1	CHX 2	CHX 3	CHX 4
lactic acid	116.4421455	122.764541	135.746381	140.461227
pyruvic acid	52.79535178	63.4095206	61.8444506	45.4579777
glycerol	3286.177048	3066.10822	3276.38016	2390.14977
glyceric acid	1.963921474	1.80608682	1.67637229	1.95621808
citric acid	170.2571405	173.569202	214.016473	178.998425
aconitate	0.988610584	1.23309464	1.12888993	1.20214519
isocitric acid	4.68814212	4.95560798	4.80801823	4.23234586
2-ketoglutaric acid	0	0	0	0
succinic acid	16.23449309	19.5620689	21.3038492	21.3693726
fumaric acid	36.73818794	44.2597738	46.771138	45.0367301
malic acid	51.64936148	60.9965654	64.3303481	61.8071522
2-hydroxyglutarate	0.149621557	0.13066544	0	0.25334465
2-aminoadipic acid	2.616714786	3.75542167	4.80216906	4.99635384
lysine	38.1900712	44.0758743	52.0634675	45.2890813
valine	931.42855	1149.17355	1354.35791	1263.08203
leucine	833.8464788	916.114792	1214.048	1094.66745
isoleucine	611.7183153	644.818484	846.935337	819.814335
threonine	37.51954496	50.0448654	52.0892039	50.1304478
homoserine	9.038250352	10.1619	9.36218247	10.6563713
glycine	33.67704172	36.6134255	45.6867017	41.95288
serine	143.0404251	170.90653	154.675467	131.672651
alanine	4.152829438	6.10933547	5.54969307	6.04947276
glutamic acid	207.5738651	292.183421	373.477732	382.485839
glutamine	101.0410999	121.385295	176.320908	161.095403
proline	797.1825475	856.763643	964.240453	1000.93489
aspartic acid	1007.674587	1168.20328	1227.20045	1021.11305
asparagine	0.919895499	1.11597969	1.45059431	1.39985336
methionine	106.3432446	128.916464	142.827387	121.904476
cysteine	102.0086527	128.134407	134.610473	129.947921
homocysteine	0.708208703	0.89239659	1.41432945	1.34918443
phenylalanine	206.0887326	255.641619	331.472498	314.315265
tyrosine	35.06685973	47.7199883	60.7366177	60.5245329
tryptophan	102.3721776	129.500103	158.849436	160.408889
histidine	6.953523324	9.29660443	5.10398627	0.32984479
4-hydroxyproline?	7.547576321	8.27838185	11.5813578	10.3175849
ornithine	72.56313023	80.2034179	67.4046722	63.5388374
phosphate	31885.92095	29390.3075	32241.6473	28453.033
diphosphate	348.842106	328.340969	483.845732	436.8536
1-phosphoglycerol	116.2714661	149.933275	254.653001	250.336303
3-phosphoglycerate	108.670691	114.098034	112.27366	111.396138
2-phosphoglycerate	6.177707843	5.1017597	6.57914709	6.14783009
DHAP	0.364633276	0.59138212	0.59544557	0
phosphoenolpyruvate	5.763200715	3.95383957	3.77505471	4.79566514

fructose	1.162614913	1.32407651	1.60618225	1.45847036
ribose	7.933267445	7.04141564	4.8513021	4.34163179
galactose or isomer	0.981960737	1.19437896	1.12538042	0.93687844
glucose	13.34291796	15.2646276	75.2355418	16.7217402
glucose-6-phosphate	35.95239769	38.3527278	34.1954212	30.7351764
mannitol	0.509821602	0.87110297	1.24002417	1.28162586
sorbitol	3.624166603	5.69314183	7.41206899	5.28844531
inositol	264.5541873	318.76174	473.517266	434.402614
myo-inositol phosphate	11.40448757	20.5057638	15.155201	11.6667694
sedoheptulose	1.674653131	2.5010334	2.34551741	2.24433552
sedoheptulose-7-P	14.36256117	19.2717013	17.4831709	17.9457426
myristic acid	16.02723953	15.0894391	15.1645597	12.7715507
palmitiladic acid	2.843917891	2.47296453	2.57363506	2.44403071
palmitic acid	745.7282482	709.527884	922.15684	721.269229
linoleic acid	0.997477047	1.12081915	1.30202538	1.17233993
oleic acid	40.35459639	37.3045006	38.1155353	40.3781691
elaidic acid	11.56186728	13.0588013	12.7149271	13.456078
stearic acid	340.0532249	331.298848	450.275002	345.761793
1-monooleoylglycerol	3.557668134	3.25405352	3.85109392	2.77983656
1-monostearylglycerol	5.572571768	6.02803252	6.00124904	7.4582677
1-monopalmitoylglycerol	26.94739657	30.6512097	31.8323562	28.2851847
2-monostearylglycerol	2.595656937	2.23099148	2.26245919	2.47582298
cholesterol	715.1034862	672.335659	960.360113	929.819561
xanthine	26.12170724	27.3884451	25.9083163	34.6297294
hypoxanthine	206.7193598	232.484799	189.98691	205.81421
adenosine	31.83392579	36.599875	26.7342192	27.8122747
adenine	116.3302064	129.600763	113.993316	150.67648
uracil	26.02417615	31.7313774	24.2272646	32.0485946
adenosine-5'-monophospha	132.0648527	187.279394	201.964842	196.703738
ribose(xyl)-5-phosphate	17.60214495	25.9017627	23.9441648	16.2150509
phosphoethanolamine	10.72176995	16.9613426	21.0675427	20.5825139
4-aminobutyrate	7.547576321	8.27838185	11.5813578	10.3175849
B-alanine	43.48999924	61.5269704	73.7545318	72.4684911
pantothenic acid	10.10444248	14.0441156	19.5502678	18.7981728
creatinine	12.57486064	12.9736268	16.791799	16.4395838
3-hydroxybutyrate	0.502063447	0.71333654	0.63054059	0.71830659
N-methylalanine	10.38041113	10.4987265	11.1485192	11.5137691
sn-glycero-3-phosphoethan	1.569363887	1.93481648	1.71146732	1.90654266
nicotinic acid	55.56279643	59.1798318	51.4422856	52.3340495
1-methylnicotinamide	35.25748868	40.3911087	39.1859335	36.6763568
cytosine	4.385574082	6.33388645	4.06166407	5.44144561

CHX 5	CHX 6	DMSO 1	DMSO 2	DMSO 3	DMSO 4	DMSO 5
147.929518	116.10042	71.5953453	69.2957727	72.203743	75.1761166	92.2003957
66.2854254	3.89059838	48.8728557	39.0247271	53.8758806	50.6580908	51.5939123
3704.17216	1843.9029	3259.26081	2251.00142	3096.56294	1750.53235	3820.22633
1.5645384	2.7063732	1.36230145	1.29558367	1.41884869	1.57010789	1.5595411
218.463319	121.128143	203.219281	259.27104	194.748553	324.600941	332.95659
1.84712022	1.03694465	0	1.03064409	1.56654287	2.38802456	2.05810519
6.90602411	3.10261016	4.57593565	4.29920275	3.74158573	7.09185682	7.83846005
0	0	0	0	0.72665533	0	0.6642993
28.0679567	23.2531286	44.9233458	44.6855058	39.9778589	44.211317	49.9678055
49.792296	29.1488355	61.6365724	73.5775069	79.1847542	75.0454771	73.1503567
71.020394	45.4379127	117.42107	121.607268	135.593098	131.339728	137.840067
0.21503788	0.22802316	0.57752267	0.61236951	0.8103487	0.73677414	0.64528051
5.4820874	2.88505364	7.21321153	8.37131443	7.93117712	8.08828705	7.01385948
62.5222626	34.4726155	107.124633	106.347525	119.856776	110.521801	102.44745
2130.43262	1262.93204	4580.09474	3133.4123	3229.61668	3049.25501	3172.36471
2005.55213	1218.1326	4475.89032	2757.77941	2941.24873	2804.4261	2950.62187
1399.51337	825.519335	3044.53882	1996.33722	2141.53007	1964.8963	2093.68936
93.4380926	45.913397	201.802255	151.350376	157.798424	126.782764	135.282039
12.4680615	10.6782123	9.16118618	7.61434419	8.30238181	7.63470033	8.43483508
59.5337875	30.9662922	131.845165	113.443636	112.558714	101.656169	104.822082
288.350629	101.817946	402.691652	228.139183	287.661973	235.881325	306.269147
8.1424919	5.48975094	19.9780926	15.2218951	16.1341115	1.64313616	15.8086932
473.30388	202.113745	800.971543	806.034523	816.729087	708.989318	707.128266
202.969565	231.285756	493.579281	389.989125	403.960303	461.852713	372.182853
1571.68703	815.519211	2915.71633	1794.21064	1912.04188	1974.54658	1749.49538
1422.00274	524.102627	1745.00104	1774.02574	2051.01422	1858.98827	2048.0592
1.7919823	1.23057743	3.92040085	4.91739511	4.37962456	4.13177745	2.98595064
228.044911	73.7718458	481.299939	377.493293	424.533117	355.534906	394.260956
125.145152	17.3731217	157.839506	353.42358	200.791214	314.593633	141.052885
1.31366087	1.24029645	2.19249029	2.71441763	2.03916422	2.01476894	1.19818401
514.083883	254.005834	1015.87751	806.125748	849.865752	682.081644	795.7966
91.5509974	68.2514426	181.797382	172.224315	160.876371	152.952851	136.497884
229.034637	134.242839	508.080224	469.514671	475.351728	429.891483	391.417646
9.65602772	3.83153664	36.3292031	29.3355081	16.180389	28.6498033	10.612487
11.8436246	7.91053448	23.3302856	20.623557	21.9473542	20.1558036	16.1225033
72.9364366	33.7728461	68.3584239	62.3432922	76.3559185	72.4643334	77.6184148
36150.1759	19170.6103	34776.722	30218.2955	30727.4193	28953.5774	39528.5919
498.683863	227.226197	383.109442	314.833626	300.372533	277.584526	360.58003
204.725707	166.735018	32.3296257	55.9595099	42.4187513	52.7369623	30.2045616
77.9002276	89.8478521	38.9932593	38.1532408	60.9868628	58.7220351	42.3874576
4.32419114	5.71553125	1.60448838	2.01567587	3.03462295	2.60305225	2.13961431
0.52794556	0.01869042	0	0	0.01181553	0.04138269	0
2.20965202	1.75465688	1.34483605	1.98170926	3.10650078	2.16893974	1.53101291

1.54661857	1.12890153	2.06441066	2.58049212	3.70023136	1.71941014	1.59893717
4.30351442	6.14615858	22.552493	12.9325453	32.5596729	25.7416551	23.9826991
0.60789554	1.27543444	0.89073556	1.05781738	1.48186488	1.28367477	1.20361796
9.91104058	14.6615151	3.41623287	3.36754707	7.17498295	4.03765212	7.92812008
22.0055427	19.4208444	0.6671784	1.18786099	1.27213915	2.13161417	1.48346592
0.90977563	0.44857014	2.20064081	2.18356799	1.91017798	2.5884466	2.13282188
4.72531949	3.86667464	9.8423369	11.6563712	7.04698134	11.9612198	13.4557967
544.151968	316.478946	525.642269	557.923942	540.053592	507.668218	475.937166
11.5376091	8.10042918	9.75966733	7.49594628	8.75924912	11.6593696	13.980172
1.25576606	1.24777262	1.08401936	0.74338359	2.17504286	1.40701141	1.46988107
11.964928	11.5177861	27.084183	20.6429665	48.4771663	31.8573674	25.8506163
15.8287175	9.27867342	20.2901411	19.2348078	24.5910799	17.8635272	26.709179
2.69900104	2.28322203	3.52801145	3.17927499	4.62282763	3.16780424	3.32964741
720.123251	693.757836	645.104462	949.49397	725.975941	693.7045	730.740098
0.97594113	1.03245895	1.22956439	1.16748102	1.83239238	1.31207465	1.18459916
32.7657072	29.76861	40.7316491	37.7048815	58.4740926	37.078889	43.4294158
10.8056533	10.0741378	12.8894676	10.6082585	17.4732054	11.0540464	12.9898362
350.089933	323.836991	294.307181	477.110576	336.764378	323.156604	348.379474
3.12769834	2.21668413	4.0007417	2.82796316	3.89912618	3.00795346	4.31047378
6.10376741	4.80418624	5.63201036	5.80149753	7.15824428	5.85362187	6.12541011
28.4883833	21.0723301	32.5461967	24.2919512	32.3341931	27.2289976	31.9257625
0	1.64400958	2.83638148	1.41883395	3.6874312	3.06231895	1.58942777
775.611294	652.707687	377.258532	468.193855	464.450914	347.169111	420.361533
27.2160758	68.1916332	28.6153167	23.7591607	37.8431858	34.4368883	27.2743089
190.658646	280.274849	756.527913	648.756488	1244.95948	1104.87721	812.182649
25.9575529	33.1904525	109.518558	69.9654002	186.445186	147.520359	111.571037
140.416976	146.531418	97.082026	109.857732	116.060051	135.902372	118.093125
32.4652055	32.2185506	18.5063413	19.6841376	27.6709957	28.6311405	33.0682486
246.948946	127.624187	155.433938	186.656244	137.945374	165.86993	149.429305
20.6505284	4.24795926	18.5855178	10.8741686	22.7015791	20.6783615	22.9665521
16.9273405	19.9643619	15.0027814	14.2620956	13.9255913	16.3664476	11.3501445
11.8436246	7.91053448	23.3302856	20.623557	21.9473542	20.1558036	16.1225033
93.971552	65.6833785	245.209604	201.601554	199.727816	225.581092	168.943946
22.8381252	19.9658571	52.6139451	53.4479215	50.6207011	50.1939555	42.3983255
26.1022899	3.18709087	52.4066889	52.9985917	54.2923782	39.1131321	34.9171471
0.80087824	0.78499775	1.62078942	1.85651803	1.54881957	1.62366196	1.71440842
12.8622976	8.92505062	23.2825469	15.6605496	16.0317102	14.3468101	16.8248402
3.5522603	0.45006538	1.40421842	1.07722687	2.50981632	0.72054564	3.51983535
57.6563414	48.9681597	41.5897825	34.2655193	48.7459697	39.3338398	39.7628641
51.4078369	40.429627	29.8588534	23.4913097	34.855825	30.2523682	37.1206102
5.25877884	3.80013673	1.93400232	1.83031521	2.57874027	2.57221809	2.6938763

DMSO 6

84.2406176
53.8882847
2254.07063
1.47893532
262.73666
1.12002649
5.32108804
0
47.6598212
81.6041291
145.683308
0.45705548
7.93833206
137.158982
2906.62942
2688.65149
1909.70001
110.748873
7.64292988
100.452135
211.642668
4.01054158
784.724812
371.165616
1691.49402
2033.53318
4.05576602
315.019958
225.49193
1.6482864
620.368623
133.71519
348.768935
29.7865463
18.007986
60.4766192
32252.7475
347.9828
50.6504074
50.2828386
2.44500627
0
1.30284868

2.24390186
27.6571489
1.20277758
18.714257
2.21792187
2.40555517
16.2067063
489.955779
9.34702517
2.40074406
33.112948
21.0717011
3.21189726
822.932726
1.41735311
40.2285802
11.6361515
382.275432
2.75869067
5.72810797
29.7345863
2.40459295
350.890635
29.9991974
857.937402
108.774394
99.0097641
22.6199164
195.991164
18.705597
14.7604865
18.007986
155.613439
45.8575793
36.8810096
1.77241305
13.6914578
2.02451523
37.3351785
35.0402788
2.36610406
